# Synergistically Enhanced Mucoadhesive and Penetrable Polypeptide Nanogel for Efficient Drug Delivery to Orthotopic Bladder Cancer

**DOI:** 10.34133/2020/8970135

**Published:** 2020-08-03

**Authors:** Hui Guo, Faping Li, Heping Qiu, Weiguo Xu, Pengqiang Li, Yuchuan Hou, Jianxun Ding, Xuesi Chen

**Affiliations:** ^1^Key Laboratory of Polymer Ecomaterials, Changchun Institute of Applied Chemistry, Chinese Academy of Sciences, 5625 Renmin Street, Changchun 130022, China; ^2^Department of Urinary Surgery, The First Hospital of Jilin University, 71 Xinmin Street, Changchun 130021, China

## Abstract

Intravesical chemotherapy has been recommended after the gold standard of transurethral resection of the bladder tumor to prevent bladder cancer (BC) from local recurrence in the clinic. However, due to rapid urine excretion and barrier protection of the bladder wall, the clinical performances of chemotherapeutic drugs are severely compromised. In the present work, a smart positively charged disulfide-crosslinked nanogel of oligoarginine-poly(ethylene glycol)–poly(L-phenylalanine-*co*-L-cystine) (R_9_-PEG–P(LP-*co*-LC)) was prepared to prolong the retention period and enhance the penetration capability of chemotherapeutic agent toward the bladder wall. PEG significantly improved the aqueous dispersibility of the 10-hydroxycamptothecin (HCPT)-loaded R_9_-PEG–P(LP-*co*-LC) (*i.e.*, R_9_NG/HCPT) and enhanced the mucoadhesive capability by the nonspecific interaction between PEG chain and the bladder mucosa accompanied with the electrostatic interaction between the cationic R_9_ and negatively charged bladder mucosa. Besides, R_9_, as a cell-penetrating peptide, efficiently penetrated through the cell membrane and delivered carried cargo. The disulfide bond endowed the selective release behavior of HCPT triggered by the intracellular reductive microenvironment. As an advanced chemotherapeutic nanoformulation, the smart R_9_NG/HCPT demonstrated superior cytotoxicity against human BC 5637 cells *in vitro* and remarkably enhanced tumor suppression activity toward orthotopic BC models of mouse and rat *in vivo*, indicating its great potential in the clinical intravesical BC chemotherapy.

## 1. Introduction

Costs of curing bladder cancer (BC) are the highest on a per-patient basis because of the elevated rate of recurrence and multiple therapeutic interventions [1]. Over 75% of BC cases are initially diagnosed with non-muscle-invasive bladder cancer (NMIBC) [2]. The recommendation for NMIBC patients after transurethral resection of the bladder tumor is immediate intravesical chemotherapy, which has been proven to help prevent cancer from recurrence [3]. However, the clinical performances of chemotherapeutic drugs are severely compromised by rapid urine excretion and barrier protection of the bladder wall. Approximately 75% of patients will experience tumor relapse or progression within five years, which significantly affects their survival [4]. The five-year survival rate of BC patients has not been improved in the past three decades [5].

Nanomedicine, as revolutionary manufacturing technology, is expected to provide great opportunities to upregulate the exposure periods of drugs within the bladder. Currently, the leading materials, such as poly(ethylene glycol) (PEG) [6], chitosan [7], and liposomes [8], have been developed to improve the mucoadhesiveness or penetrability. PEG seems to be the most promising mucoadhesive polymer due to the nonspecific interaction between PEG chain and mucous membrane [9]. Moreover, PEG possesses the capability of improving the water solubility of hydrophobic drugs [10] and is well known for its reduced toxicity and low immunogenicity [11]. Cell-penetrating peptides (CPPs) are potentially less cytotoxic and possess the capacity of permeating through cell membrane to transport carried cargoes [12, 13]. The combination of CPPs with anti-tumor drugs has been considered an incredibly attractive concept to improve therapeutic efficacy [14]. Among them, the arginine-rich CPPs have been demonstrated to be highly efficient in terms of cell uptake, especially the oligoarginine of nine arginine residues (R_9_) [15, 16].

**Scheme 1:**
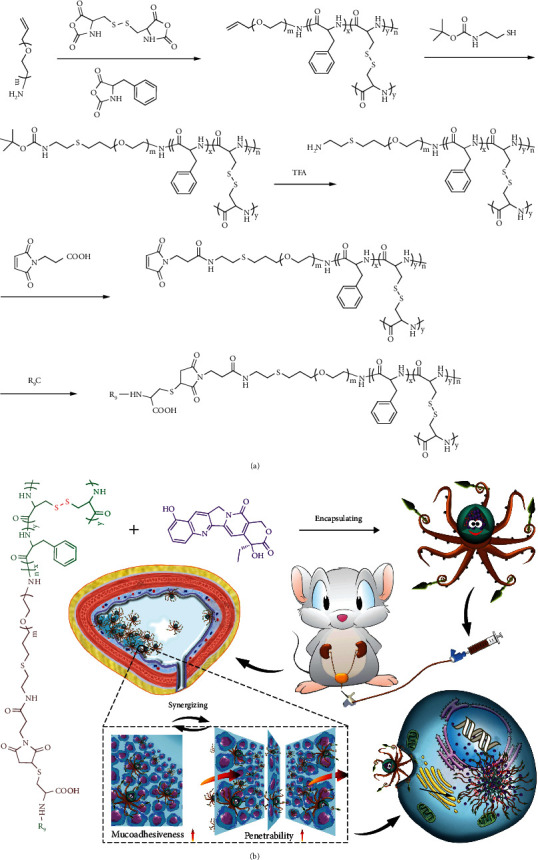
Synthesis and *in vivo* metabolism of water-soluble mucoadhesive nanogel. (A) Synthesis route of R_9_-PEG–P(LP-*co*-LC). (B) Schematic illustration for self-assembly of R_9_NG/HCPT, intravesical chemotherapy of R_9_NG/HCPT, superior mucoadhesiveness, enhanced penetrability, selective accumulation in tumor tissue, promoted internalization, and accelerated HCPT release from R_9_NG/HCPT.

Accordingly, an innovative nanogel of R_9_-poly(ethylene glycol)–poly(L-phenylalanine-*co*-L-cystine) (R_9_-PEG–P(LP-*co*-LC)) was successfully synthesized to improve the mucoadhesiveness and penetrability of chemotherapeutical drugs (Scheme 1A). 10-Hydroxycamptothecin (HCPT) was applied as a model drug and encapsulated into the core of R_9_-PEG–P(LP-*co*-LC) by facile diffusion. The obtained drug-loaded nanogel was referred as R_9_NG/HCPT. The morphology of R_9_NG/HCPT is similar to that of an octopus with spears in its hands, as shown in Scheme 1B. The rinsing of R_9_NG/HCPT caused by urine excretion was resisted by the presence of PEG chains, which were like arms of an octopus, and R_9_, which enhanced the mucoadhesion of R_9_NG/HCPT through the nonspecific and electrostatic interactions, respectively. The powerful penetrating capacity of R_9_ overcame barrier protection of the bladder wall. R_9_ was the weapon in the hands of octopus to break through obstacles. The drug-loaded core of polypeptide nanogel was much like the head of the octopus, which released the cargo triggered by the high concentration of glutathione (GSH) in BC cells [17].

The mucoadhesive nanogel (*i.e.*, PLL–P(LP-*co*-LC)) reported previously was synthesized by the ring-opening polymerization (ROP) of *N*(*ε*)-benzyloxycarbonyl-L-lysine *N*-carboxyanhydride (ZLL NCA), L-phenylalanine *N*-carboxyanhydride (LP NCA), and L-cystine *N*-carboxyanhydride (LC NCA) [18]. The nanogel reported in this work, as distinct from PLL–P(LP-*co*-LC), introduced PEG and R_9_ and further magnified the mucoadhesion and penetration of R_9_NG/HCPT as a benefit of the synergistic effect of PEG and R_9_.

The nanogel composed of PEG and polypeptide, but without R_9_, was synthesized as a negative control (*i.e.*, NG/HCPT). In order to better understand the anti-tumor efficacy of R_9_NG/HCPT *in vivo*, different orthotopic BC models of mouse and rat were constructed. The orthotopic BC model in mouse was constructed by instilling MB49 cells into the bladder that was pretreated with PLL solution [19]. The orthotopic BC model in rat was successfully established by intravesical instillation of *N*-methyl-*N*-nitrosourea (MNU). MNU played a vital role in the process of carcinogenesis, which was histologically indistinguishable from the human tumor [20]. The physicochemical properties, intracellular release profiles, and cytotoxicity of R_9_NG/HCPT *in vitro* and mucoadhesiveness, permeability, biodistribution, anti-tumor activity, and systemic toxicity *in vivo* were systematically demonstrated. Encouragingly, R_9_NG/HCPT possessed unique physicochemical properties to improve the chemotherapy efficacy of BC both *in vitro* and *in vivo*, as a potential benefit of strong urothelium adhesive property, high penetrating capacity, and selective drug release behavior.

## 2. Results and Discussion

### 2.1. Preparation and Characterizations of Empty and HCPT-Loaded R_9_-PEG–P(LP-*co*-LC)

aPEG–P(LP-*co*-LC) was synthesized by the ROP of LP NCA and LC NCA with the amino-terminated allyloxy poly(ethylene glycol) (aPEG-NH_2_) as a macroinitiator. R_9_-PEG–P(LP-*co*-LC) was synthesized by the decoration of R_9_ at the end of PEG by sequential deprotection, amidation, and Michael addition reactions, as depicted in Scheme 1A. The chemical structures of intermediates and the desired product R_9_-PEG–P(LP-*co*-LC) were confirmed by the proton nuclear magnetic resonance (^1^H NMR) spectra. The characteristic resonance signals at 7.19 ppm (e; –CH_2_C_6_*H*_5_, 5H) and 3.46 ppm (d; –C*H*_2_C_6_H_5_, 2H) were assigned to the protons in the L-phenylalanine unit. The peak at 2.42 ppm (g; –SC*H*_2_–, 2H) was ascribed to protons of the L-cystine unit. The signal at 4.55 ppm (c+f; –COC*H*(CH_2_)NH–, 1H) was attributed to the protons of peptide backbone both in the polypeptide and R_9_. The strong and broad signal peak of b at 3.82 ppm (b; –OC*H*_2_C*H*_2_–, 4H) was assigned to the methylene protons of PEG. The signal at 2.42 ppm (a; –SC*H*_2_CH_2_CH_2_–, 2H) was produced by the thiol-ene click reaction between allyloxy and thiol groups. The peaks at 5.28 and 5.82 ppm (a and b; C*H*_2_=C*H*–, 2H and 1H, respectively) were attributed to the allyloxy protons of aPEG–P(LP-*co*-LC), as shown in Supplementary Figure S1. The signal disappearance of the allyloxy protons (i) and the appearance of the *t*-butyloxycarbonyl (*t*-Boc) resonance signal at 1.52 ppm (j; (C*H*_3_)_3_C–, 9H) indicated the successful synthesis of *t*-Boc-NH-PEG–P(LP-*co*-LC), as depicted in Supplementary Figure S2 . The disappearance of *t*-Boc peak (j) demonstrated the successful synthesis of NH_2_-PEG–P(LP-*co*-LC), as shown in Supplementary Figure S3. The maleimide-terminated polymer was produced by a simple amidation reaction, which was confirmed by the signal at 6.48 ppm (h; –C*H*=C*H*–, 2H), as shown in Supplementary Figure S4B. The representative peaks at 1.4–4.2 ppm in Supplementary Figure S4C matched precisely to the signals in Supplementary Figure S4A , and the signal disappearance of two vinyl protons on maleimide at 6.48 ppm (h) both confirmed the successful synthesis of R_9_-PEG–P(LP-*co*-LC). The nanogel of Mal-PEG–P(LP-*co*-LC) was synthesized as a negative control. The molar ratio of LP and LC in R_9_-PEG–P(LP-*co*-LC) was calculated as 9:5 according to the content of carbon (C), hydrogen (H), nitrogen (N), and sulfur (S) determined by elemental analysis.

**Figure 1:**
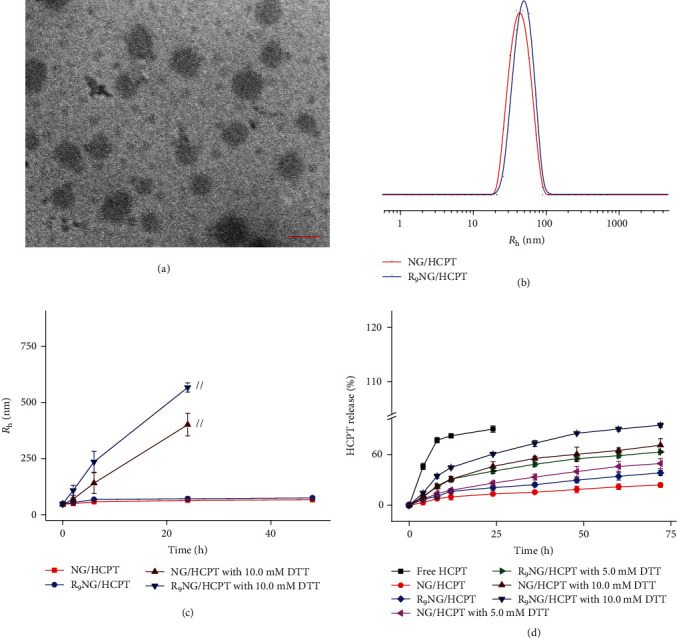
Characterizations of R_9_NG/HCPT. (a) Morphologies of R_9_NG/HCPT in PBS by TEM. (b) *R*_h_ of R_9_NG/HCPT in PBS. (c) Stability of R_9_NG/HCPT in PBS without or with 10.0 mM DTT versus time. The scale bar in (a) represents 100 nm. // represents the interruption of *R*_h_-related light-scattering signal. (d) *In vitro* release profiles of R_9_NG/HCPT. All the statistical data are represented as mean ± standard deviation (*n* = 3).

The drug loading contents (DLCs) of R_9_NG/HCPT and NG/HCPT were up to 26.2 ± 0.3 and 25.6 ± 0.4 wt.%, respectively. The drug loading efficiencies (DLEs) of R_9_NG/HCPT and NG/HCPT were 88.9 ± 1.4 and 86.0 ± 1.6 wt.%, respectively. As depicted in Figure 1(a), the resulting R_9_NG/HCPT displayed spherical morphology, which was detected by transmission electron microscopy (TEM). The hydrodynamic radius (*R*_h_) of R_9_NG/HCPT in phosphate-buffered saline (PBS) at pH 7.4 was 48.9 ± 0.7 nm, while that of NG/HCPT was detected to be 44.8 ± 1.3 nm (Figure 1(b)). Even when incubated with PBS for 48 h, sizes of R_9_NG/HCPT and NG/HCPT almost maintained constant. However, after co-incubation with 10.0 mM D,L-1,4-dithiothreitol (DTT) for 24 h, the *R*_h_ of NG/HCPT increased to 401.0 ± 50.3 nm, and that of R_9_NG/HCPT increased to 566.7 ± 20.6 nm, which could not be detected after 24 h (Figure 1(c)). The results demonstrated the reduction-responsive properties of NG/HCPT and R_9_NG/HCPT, which were critical for the selective delivery of drugs to cancer cells.

The zeta potential of NG/HCPT was −17.3 ± 0.8 mV, while that of R_9_NG/HCPT was 26.9 ± 1.2 mV. The positive charge of R_9_NG/HCPT further demonstrated the successful conjugation of R_9_ onto the surface of the nanogel, which could enhance the interaction between R_9_NG/HCPT and negatively charged cell membrane and subsequently upregulated cell internalization [21]. The release behavior of HCPT from R_9_NG/HCPT was examined in PBS at pH 7.4 with 0.1% (*W*/*V*) Tween-80 containing different concentrations of DTT. As shown in Figure 1(d), free HCPT displayed a rapid diffusion from the dialysis membrane within 12 h. In contrast, R_9_NG/HCPT released only 38.2% of HCPT throughout 72 h in PBS without DTT, demonstrating excellent stability of R_9_NG/HCPT under physiological conditions. In addition, the release rate of HCPT from R_9_NG/HCPT was remarkably accelerated due to the increased concentration of DTT. The cumulative release amount of HCPT was 62.9% and 94.5% over a period of 72 h with the concentration of DTT ranging from 5.0 to 10.0 mM, respectively. The results demonstrated the excellent physiological stability of R_9_NG/HCPT. However, the disulfide bond could be rapidly broken in the reductive microenvironment, resulting in the selective HCPT release in the cancer cells.

### 2.2. Intracellular Release and Cytotoxicity of R_9_NG/HCPT

**Figure 2:**
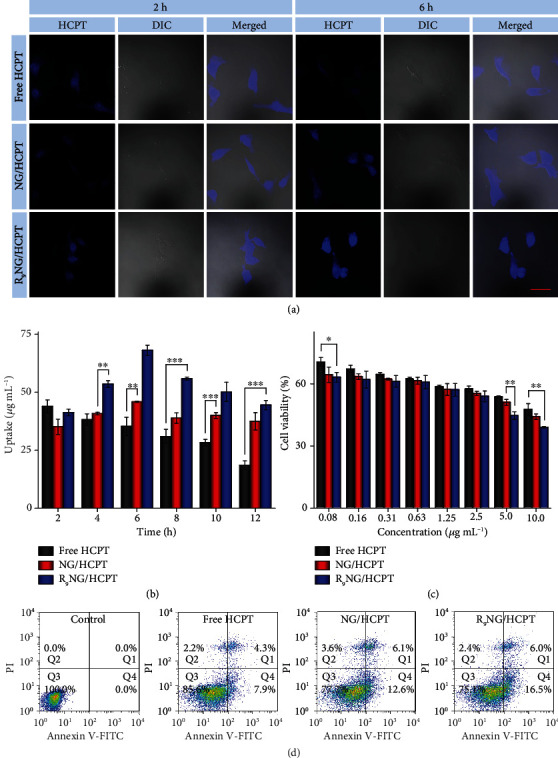
*In vitro* cell uptake and cytotoxicity. (a) Representative CLSM images of human BC 5637 cells incubated with free HCPT, NG/HCPT, or R_9_NG/HCPT for 2 or 6 h. The blue color was HCPT fluorescence in cells (left). Differential interference contrast (DIC) was used to obtain the bright-field images (middle), and the merged images were shown on the right. The scale bar represents 50 *μ*m. (b) Intracellular HCPT release profiles in human BC 5637 cells after co-incubation with free HCPT, NG/HCPT, or R_9_NG/HCPT. Data are presented as mean ± standard deviation (*n* = 3; ^∗^*P* < 0.05, ^∗∗^*P* < 0.01, and ^∗∗∗^*P* < 0.001). (c) The viability of human BC 5637 cells incubated with free HCPT, NG/HCPT, or R_9_NG/HCPT at different concentrations for 24 h. Data are presented as mean ± standard deviation (*n* = 3; ^∗^*P* < 0.05, ^∗∗^*P* < 0.01). (d) Apoptotic cell populations were calculated by FCM analysis after co-incubating human BC 5637 cells with PBS as a control, free HCPT, NG/HCPT, or R_9_NG/HCPT for 24 h. The lower-left (Q3), lower-right (Q4), upper-right (Q1), and upper-left (Q2) quadrants in each panel indicated the populations of healthy, early and late apoptotic, and necrotic cells, respectively.

The intracellular release behavior of R_9_NG/HCPT was qualitatively evaluated by confocal laser scanning microscopy (CLSM).  Figure 2(a) presents the CLSM images of human BC 5637 cells after co-incubation with free HCPT, NG/HCPT, or R_9_NG/HCPT with equivalent HCPT concentration at 1.25 *μ*g mL^−1^. The cells depicted slightly weaker HCPT fluorescence after co-incubation with R_9_NG/HCPT for 2 h as compared with that of free HCPT. At the same point, the fluorescence intensity of negatively charged NG/HCPT was the weakest. Although it was not as simple as small-molecule drugs entering cells by diffusion, the results demonstrated that R_9_NG/HCPT could be internalized by human BC 5637 cells effectively [22]. Furthermore, after the co-incubation period increased up to 6 h, the cells treated with R_9_NG/HCPT exhibited more robust HCPT fluorescence than that of free HCPT or NG/HCPT (Figure 2(a)). The significantly enhanced HCPT fluorescence mainly benefited from the electrostatic interaction between the positively charged R_9_NG/HCPT and the negatively charged cell membrane [23]. The conjugated R_9_ facilitated the cell uptake of R_9_NG/HCPT through the CPP-mediated cell entry [24]. In addition, the high concentration of GSH in human BC 5637 cells led to the rupture of the disulfide bond and accelerated the HCPT release from R_9_NG/HCPT [25].

The cell uptake and subsequent release profiles were investigated quantitatively according to the protocol described by Wei et al. [26]. Compared with free HCPT, the cells co-cultured with R_9_NG/HCPT displayed a slightly lower content of HCPT at 2 h. The accumulation of HCPT in cells co-cultured with NG/HCPT was the lowest at the same time point. With the prolongation of co-incubation time, the endocytosis of R_9_NG/HCPT increased significantly, and a relatively higher content of HCPT was maintained as compared to that of free HCPT or NG/HCPT from the second time point. Conversely, the increased HCPT concentration in the cells of the R_9_NG/HCPT group was about 1.9 times compared with that in the cells treated with free HCPT (Figure 2(b)), while it was about 1.5 times as much as that in the NG/HCPT group at 6 h. The results were consistent with the representative finding by CLSM (Figure 2(a)).

To evaluate the potential cytotoxicity of R_9_NG/HCPT against human BC 5637 cells, a standard methyl thiazolyl tetrazolium (MTT) assay was performed. The cytotoxicity of free HCPT, NG/HCPT, and R_9_NG/HCPT was enhanced in a concentration-dependent manner (Figure 2(c)). Furthermore, the cytotoxicity of R_9_NG/HCPT surpassed those of free HCPT and NG/HCPT against human BC 5637 cells. The superior cytotoxic effect of R_9_NG/HCPT probably benefited from the weak positive surface, R_9_, and the reduction-responsive property of R_9_NG/HCPT, which led to the promoted cell uptake and accelerated intracellular HCPT release in human BC 5637 cells. Importantly, the half-maximal inhibitory concentration (IC_50_) of R_9_NG/HCPT was 2.4 ± 0.1 *μ*g mL^−1^, which was lower in comparison with those of free HCPT (*i.e.*, 8.7 ± 0.8 *μ*g mL^−1^) and NG/HCPT (*i.e.*, 4.7 ± 0.2 *μ*g mL^−1^). The lowest IC_50_ indicated the potent cytotoxicity of R_9_NG/HCPT quantitatively, suggesting the superiority of R_9_NG/HCPT for intravesical chemotherapy of BC *in vivo*.

The R_9_NG/HCPT-induced apoptosis of human BC 5637 cells was measured by flow cytometry (FCM) analysis. As shown in Figure 2(d), the 24 h exposure to three different formulations of HCPT at an equivalent HCPT dosage of 0.1 *μ*g mL^−1^ resulted in a noticeable difference in the percentage of apoptotic cells. R_9_NG/HCPT contributed most to an abundance of apoptotic cells. The total percentage of apoptotic cells was 22.5%, which was constituted with 16.5% early apoptotic cells and 6.0% late apoptotic cells. In contrast, the total percentage of cells displaying apoptosis was reduced to 12.2% and 18.7% after co-incubation with free HCPT and NG/HCPT, respectively. The results were consistent with the cytotoxicity of R_9_NG/HCPT described above. When the co-incubation time was prolonged to 48 h, the differences among free HCPT, NG/HCPT, and R_9_NG/HCPT groups varied greatly, as shown in Supplementary Figure S5. The total percentage of cells displaying apoptosis was increased to 38.9%, 53.5%, or 63.0% after co-incubation with free HCPT, NG/HCPT, or R_9_NG/HCPT, respectively. This phenomenon indicated that different formulations induced the apoptosis of human BC 5637 cells in a time-dependent manner. The increased apoptotic activity induced by R_9_NG/HCPT benefited from the high intracellular concentration of HCPT, which was internalized through CPP-mediated endocytosis and triggered to release by GSH rapidly.

### 2.3. Mucoadhesiveness, Permeability, and Biodistribution of R_9_NG/HCPT

CLSM was employed to demonstrate the mucoadhesiveness and permeability of R_9_NG/HCPT. The male Sprague-Dawley (SD) rats with BC were anesthetized and then administrated with different HCPT formulations at a consistent HCPT dosage of 6.0 mg per kg body weight (mg (kg BW)^−1^) through bladder irrigation. At different indicated time points (*i.e*., 0.5, 2, 6, 12, 24, and 48 h), the bladders were removed and sectioned.

**Figure 3:**
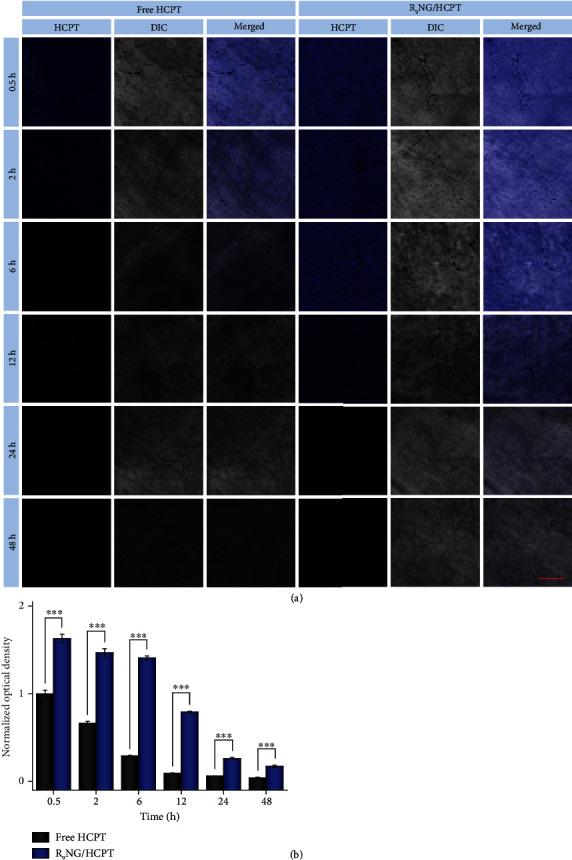
Mucoadhesiveness. (a) The fluorescence intensities of free HCPT and R_9_NG/HCPT adhered to the inner surface of the bladders were determined by CLSM. (b) Quantitative evaluation of HCPT fluorescence intensities from results by CLSM. The scale bar in (a) represents 100 *μ*m. Data are presented as mean ± standard deviation (*n* = 3; ^∗∗∗^*P* < 0.001).

As depicted in Figure 3(a), the fluorescence signals of free HCPT and R_9_NG/HCPT were observed in the bladder sections at the first few time points. The fluorescence intensity of R_9_NG/HCPT was stronger than that of free HCPT. The highest fluorescence signal was observed in the bladder treated with R_9_NG/HCPT at 0.5 h. Not surprisingly, the fluorescence intensity of R_9_NG/HCPT decreased over time. However, it remained at a relatively high concentration even at 12 h, indicating the high mucoadhesion of R_9_NG/HCPT toward the urothelial surface. On the contrary, the fluorescence signal in the bladder exposed to free HCPT decreased rapidly, which was attributed to the continuous excretion of urine. This phenomenon demonstrated the outstanding mucoadhesiveness of R_9_NG/HCPT. In order to quantitatively prove the mucoadhesiveness of R_9_NG/HCPT, the average optical density of HCPT was measured. As verified in Figure 3(b), the fluorescence intensity of R_9_NG/HCPT was 1.6 times higher as compared with that of free HCPT at 0.5 h. Inspiringly, the fluorescence intensity increased from 2.2 to 4.8 times when the retention time was prolonged from 2 to 6 h. The outstanding mucoadhesiveness of R_9_NG/HCPT benefited from the nonspecific interaction between PEG chain and the bladder mucosa and electrostatic interaction between terminal cationic R_9_ and negatively charged surface of the bladder mucosa, which retarded the wash-off of R_9_NG/HCPT by urine [27, 28].

**Figure 4:**
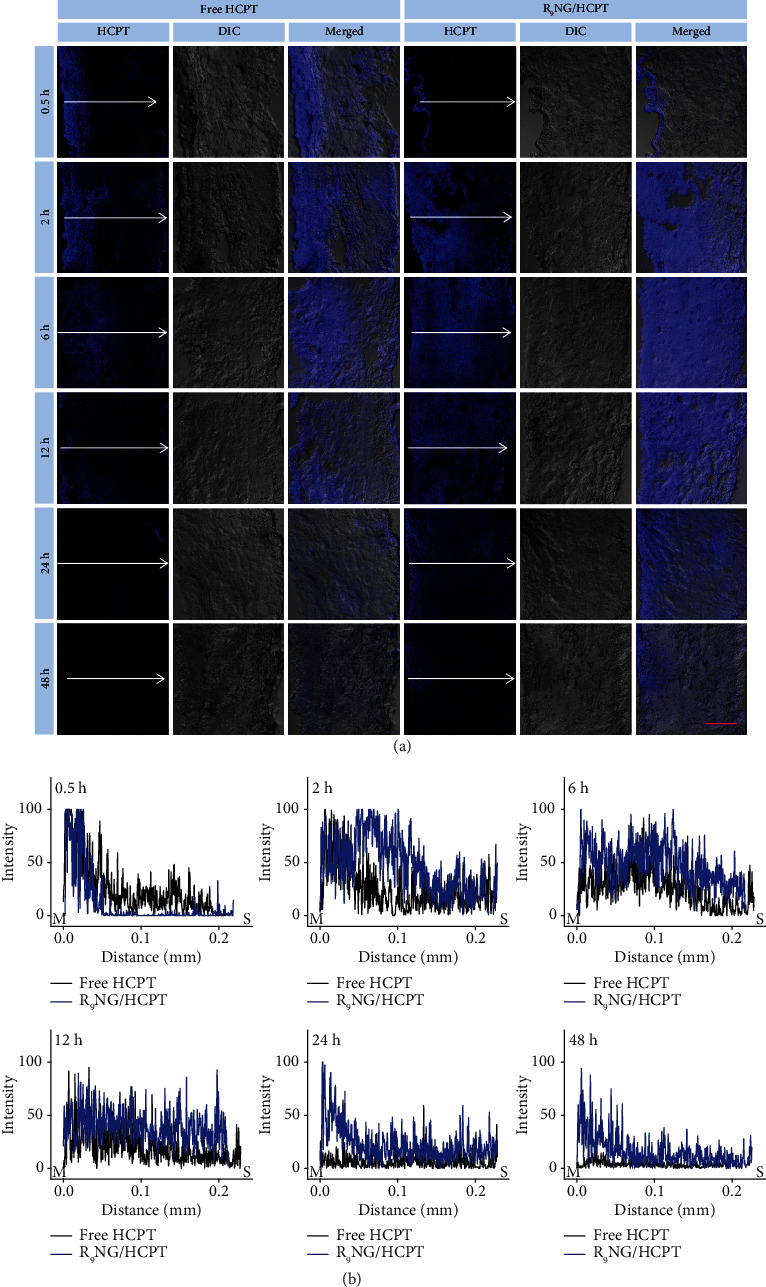
Penetrability. (a) Penetration depth of free HCPT and R_9_NG/HCPT was observed by CLSM. (b) Bladder penetration quantification of HCPT fluorescence intensity. The arrow represents the direction of HCPT penetration. The scale bar in (a) represents 100 *μ*m. M, mucous membrane; S, serous membrane.

The permeability of R_9_NG/HCPT was verified in Figure 4(a). Within half an hour, a much stronger fluorescence signal was observed in the full-thickness bladder wall treated with free HCPT and especially confined to the mucous membrane. At the same time, R_9_NG/HCPT displayed a shallow penetration within the bladder wall. However, R_9_NG/HCPT gradually penetrated through the full-thickness bladder over time. It also maintained a relatively stronger HCPT fluorescence than free HCPT for an extended period. The results indicated the exceptional permeability and accumulation of R_9_NG/HCPT in the bladder. For detailed analysis of the permeability of R_9_NG/HCPT, the HCPT fluorescence signals were normalized to the maximum value. As shown in Figure 4(b), the fluorescence intensity of R_9_NG/HCPT gradually spread throughout the whole bladder tissue over time and remained a comparatively higher HCPT content. The phenomenon was mainly due to the strong penetration ability of R_9_ with a positive surface charge [29, 30]. In addition, the advanced mucoadhesiveness of R_9_NG/HCPT further promoted its permeability. An excellent chemotherapeutic effect of R_9_NG/HCPT was predictable based on superior permeability, which was vital for the treatment of BC.

The bladder accumulation of R_9_NG/HCPT was verified by high-performance liquid chromatography (HPLC). Male SD rats were administrated with intravesical instillation of different HCPT solutions, and the experimental rats were euthanized at 6 h. Subsequently, the bladders and other major organs, such as the heart, liver, spleen, lung, and kidney, were excised and homogenized. Then, the samples were extracted with the organic phase of methanol and acetonitrile (1/1, *V*/*V*). 20.0 *μ*L of mixture suspension collected by centrifugation was analyzed for the determination of drug content. HCPT, released from R_9_NG/HCPT, was mainly accumulated in the bladder, which was 2.2 times higher as compared with free HCPT (Figure S6, Supporting Information). In addition, the other organs provided extremely low drug content. The results confirmed the advanced mucoadhesiveness of R_9_NG/HCPT and predicted an improved anti-tumor efficacy of R_9_NG/HCPT with reduced toxicity on normal organs.

### 2.4. In *Vivo* Anti-Tumor Efficacy of R_9_NG/HCPT

**Figure 5:**
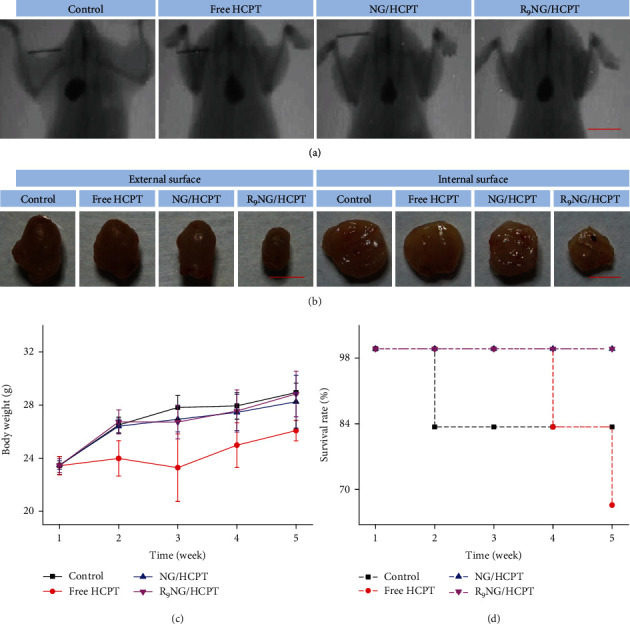
*In vivo* anti-tumor efficacy on orthotopic BC in C57Bl/6 mice. (a) Cystography and (b) external and internal surface of bladders after intravesical chemotherapy with PBS as a control, free HCPT, NG/HCPT, or R_9_NG/HCPT. (c) Evolution of body weight and (d) survival rate during the experiments. The scale bars represent (a) 1.0 cm and (b) 0.5 cm, respectively. Data are presented as mean ± standard deviation (*n* = 6).

The anti-tumor efficacy of R_9_NG/HCPT was assessed on the orthotopic BC in C57Bl/6 mice. Briefly, PLL (100.0 *μ*L of 0.1 mg mL^−1^ for 20 min) was used to disrupt the glycosaminoglycan layer. 50.0 *μ*L of MB49 cell suspension, including 1.0 × 10^5^ cells, was slowly injected into the bladder of C57Bl/6 mouse, and the cells were allowed to dwell in the bladder for 50 min [31]. The tumor-bearing mice were randomly divided into four groups: PBS as a control, free HCPT, NG/HCPT, and R_9_NG/HCPT (*n* = 6). Different HCPT formulations were administered weekly by catheterization at a dosage of 6.0 mg (kg BW)^−1^ for a total of four treatments. To define the development of BC, especially the tumor size, cystography was performed. Polypoid filling defects and irregular surface were manifested in cystography after treatment, which was depicted in Figure 5(a). The most extensive space-occupying lesion was identified in the bladder exposed to PBS, while there was no significant abnormality recognized in the bladder administrated with R_9_NG/HCPT based on the smooth margin. The superior anti-tumor efficacy of R_9_NG/HCPT was intuitionally approved by cystography. The result was mostly attributed to the presence of PEG and R_9_ in R_9_NG/HCPT, which could eventually result in a remarkably enhanced mucoadhesiveness and permeability to improve the anti-tumor efficacy synergistically. The suboptimal BC growth inhibition of free HCPT was caused by rapid drug excretion from urine.

The anti-tumor efficacy of R_9_NG/HCPT was further assessed on another orthotopic BC in male SD rats. The orthotopic BC was established by four doses of MNU [32]. Histopathological analysis was performed to demonstrate the successful induction of BC after the intravesical instillation of MNU for eight weeks. As verified in Supplementary Figure S7A, the abundance of cancer cells was observed with apparent architectural disarray and nuclear atypia, indicating the occurrence of BC. The BC-bearing rats were randomly divided into three groups: PBS as a control, free HCPT, and R_9_NG/HCPT (*n* = 8). The equivalent HCPT dosage of 6.0 mg (kg BW)^−1^ was given by intravesical instillation weekly for a total of six treatments. Cystography was performed to define the development of BC. As pointed in Supplementary Figure S7B, the results were consistent with those of cystography in mice (Figure 5(a)).

The bladders were harvested at the end of the treatments to assess the external surface, which was shown in Figure 5(b) and Figure S7B, Supporting Information. A rigid-walled bladder with the largest volume was detected in the PBS group, suggesting that exuberant growth of the tumor had infiltrated the muscular layer of the bladder wall. In contrast, a smooth-surfaced bladder with standard size and general shape occurred in the R_9_NG/HCPT group. The bladders exposed to free HCPT or NG/HCPT were generally less rigid, suggesting that free HCPT and NG/HCPT were effective but less effective than R_9_NG/HCPT. To be better informed of the growth of the tumor, the bladders were opened up, and the internal surface was displayed (Figure 5(b) and Figure S7B, Supporting Information). Multiple neoplasms with a “fish flesh” soft tan appearance were found in the bladder cavity of the control group, while there was no obvious abnormality in the bladder treated with R_9_NG/HCPT. The bladder observation intuitively confirmed that R_9_NG/HCPT possessed an excellent anti-tumor efficacy on BC both in mouse and rat.

In order to evaluate the systemic toxicity of R_9_NG/HCPT, the body weights of the experimental animals were monitored once a week and shown in Figure 5(c) and Figure S7C, Supporting Information. The experimental animals administrated with R_9_NG/HCPT showed a stable increase in body weight during the treatment, indicating negligible systemic toxicity of R_9_NG/HCPT. Another essential predictor of efficacies and toxicity of various drug formulations was the survival rate, which was shown in Figure 5(d) and Figure S7D , Supporting Information [33]. Different HCPT formulations extended the overall survivals of experimental animals in comparison with the control group. Intravesical instillation of R_9_NG/HCPT has shown an especially significant survival advantage in the murine orthotopic BC model. These encouraging results benefited from the excellent biochemical properties of R_9_NG/HCPT. R_9_NG/HCPT possessed improved mucoadhesiveness and permeability and could selectively release HCPT within cancer cells. All these results suggested that the tumor growth inhibition effect was enhanced, while the systemic toxicity was decreased, which confirmed the reliable biological safety of R_9_NG/HCPT.

**Figure 6:**
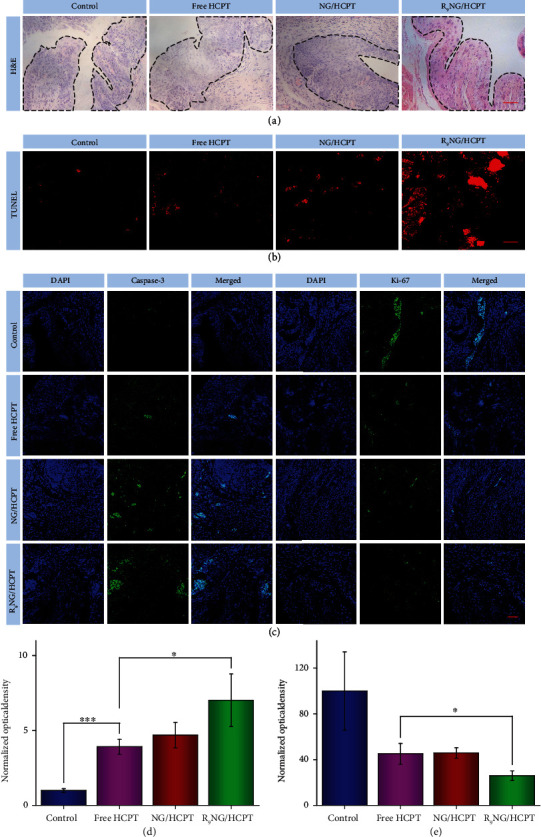
Histopathology and immunofluorescence of orthotopic BC in C57Bl/6 mice. (a) Histopathological (*i.e.*, H&E), (b) apoptosis (*i.e.*, TUNEL), and (c) immunofluorescence (*i.e*., caspase-3 and Ki-67) analysis of tumor tissue sections after treatment with PBS as a control, free HCPT, NG/HCPT, or R_9_NG/HCPT. The scale bars in (a), (b), and (c) represent 50 *μ*m. The quantitative analysis of (d) caspase-3 and (e) Ki-67 expression after treatments with different HCPT formulations. Data are presented as mean ± standard deviation (*n* = 3; ^∗^*P* < 0.05, ^∗∗∗^*P* < 0.001).

To further accurately evaluate the anti-tumor activity of R_9_NG/HCPT, the histopathological examination of the bladders was performed after the completion of intravesical chemotherapy. For hematoxylin and eosin (H&E) staining (Figure 6(a) and Figure S8A, Supporting Information), large amounts of cancer cells with spherical or spindle nuclei and prominent atypia were observed in the bladder section of the PBS group. Fortunately, the tumor cells showed karyopyknosis, chromatic agglutination, apoptotic body, and ill-defined morphology in the bladder administrated with R_9_NG/HCPT, indicating the predominant contribution of R_9_NG/HCPT in tumor suppression.

Moreover, the detection of apoptotic DNA fragments was performed using the terminal deoxyribonucleotide transferase (TdT)-mediated biotin-16-dUTP nick-end labeling (TUNEL) method. As depicted in Figure 6(b), the red fluorescence of Cy3 was observed in all the bladder sections. In particular, the bladder treated with R_9_NG/HCPT showed a larger area of flake-like red fluorescence as compared to those of the other three groups.

Caspase-3, a crucial mediator of programmed cell apoptosis, is located downstream of apoptotic response [34]. To further analyze the apoptosis mediated by R_9_NG/HCPT, immunofluorescence staining for caspase-3 was performed. The expression of caspase-3 in mouse and rat bladder tissues was depicted in Figure 6(c) and Figure S8B, Supporting Information, respectively. The level of caspase-3 was much higher in the section administrated with R_9_NG/HCPT as compared to that administrated with free HCPT or NG/HCPT. A feeble positive signal was observed in the bladder section of control group. The phenomenon indicated that a large number of cells were undergoing apoptosis in the R_9_NG/HCPT group.

Ki-67, a cell proliferative marker, is expressed from prophase to anaphase [35]. To confirm the cell proliferation status after treatment with R_9_NG/HCPT, the expression of Ki-67 in different experimental animals was monitored by immunofluorescence, as shown in Figure 6(c) and Figure S8B, Supporting Information. The strongest positive signal was recorded in the control group. The proportion of Ki-67-positive cells diminished significantly due to the use of different HCPT formulations, especially R_9_NG/HCPT, which demonstrated that the growth of BC cells was obviously suppressed by R_9_NG/HCPT.

To further confirm the expression of caspase-3 and Ki-67, the quantitative analysis was performed by a similar method as mucoadhesiveness mentioned above. The quantitative data described the order of caspase-3 expression as R_9_NG/HCPT > NG/HCPT > free HCPT > PBS as a control. More importantly, the expression of caspase-3 in the R_9_NG/HCPT group was 7.0 (Figure 6(d), BC in mouse) and 5.6 (Figure S8C, BC in rat) times higher as compared to that of the free HCPT group, which demonstrated that a large number of cells were undergoing apoptosis in the R_9_NG/HCPT group. The quantitative data of Ki-67 demonstrated that the Ki-67 signal was sorted as PBS as a control > free HCPT > NG/HCPT > R_9_NG/HCPT, demonstrating a significant proliferation inhibition of tumor cells induced by R_9_NG/HCPT (Figure 6(e) and Figure S8D, Supporting Information). All these results indicated that R_9_NG/HCPT possessed unique advantages for BC chemotherapy.

## 3. Conclusion

In summary, a cationic R_9_-decorated disulfide-crosslinked R_9_NG/HCPT with enhanced mucoadhesion and penetration toward the bladder mucosa was designed and developed for intravesical chemotherapy. PEG and R_9_ endowed R_9_NG/HCPT with upregulated mucoadhesiveness by the nonspecific and electrostatic interactions, respectively, and R_9_ further promoted the permeability of laden nanogel within the bladder wall. The advanced properties of R_9_NG/HCPT overcame the disadvantages of clinical chemotherapeutic drugs, which will bring unpredictable benefits to BC patients and increase overall survival. More importantly, the reduction-responsive property enabled R_9_NG/HCPT to release the cargo specifically in cancer cells triggered by the intracellular reductive microenvironment. This means that R_9_NG/HCPT may achieve the same intracellular drug concentration as clinical chemotherapeutic agents, but the administered quantity of R_9_NG/HCPT will decrease. The property avoided the potential risk of drug resistance due to high-dose chemotherapy, which was fundamentally critical for patients with BC. The extremely outstanding anti-tumor efficacy and the negligible systemic toxicity pave the way of R_9_NG/HCPT for further progress in the clinical applications.

## Data Availability

All data are available in the manuscript and supplementary materials, or from the authors.

## Supplementary Materials

Supplementary Materials Supplementary 1. Materials and Methods. Supplementary 2. Figure S1: ^1^H NMR spectrum of aPEG–P(LP-*co*-LC) in deuterated trifluoroacetic acid (TFA-*d*). Supplementary 3. Figure S2: ^1^H NMR spectrum of *t*-Boc-NH-PEG–P(LP-*co*-LC) in deuterated trifluoroacetic acid (TFA-*d*). Supplementary 4. Figure S3: ^1^H NMR spectrum of NH_2_-PEG–P(LP-*co*-LC) in deuterated trifluoroacetic acid (TFA-*d*). Supplementary 5. Figure S4: ^1^H NMR spectra of (A) R_9_-PEG–P(LP-*co*-LC), (B) Mal-PEG–P(LP-*co*-LC), and (C) R_9_C in deuterated trifluoroacetic acid (TFA-*d*). Supplementary 6. Figure S5: FCM analysis calculated apoptotic cell populations after co-incubating human BC 5637 cells with PBS as a control, free HCPT, NG/HCPT, or R_9_NG/HCPT for 48 h. The lower-left (Q3), lower-right (Q4), upper-right (Q1), and upper-left (Q2) quadrants in each panel indicated the populations of healthy, early and late apoptotic, and necrotic cells, respectively. Supplementary 7. Figure S6: *In vivo* biodistribution of free HCPT and R_9_NG/HCPT. Data are presented as mean ± standard deviation (*n* = 3; ^∗∗^*P* < 0.01). Supplementary 8. Figure S7: *In vivo* anti-tumor efficacy on orthotopic BC in SD rats. (A) Cystography and (B) external and internal surface of bladders after intravesical chemotherapy with PBS as a control, free HCPT, or R_9_NG/HCPT. (C) Evolution of body weight and (D) survival rate during the experiments. The scale bars represent (A) 1.0 cm and (B) 0.5 cm, respectively. Data are presented as mean ± standard deviation (*n* = 8). Supplementary 9. Figure S8: histopathology and immunofluorescence of orthotopic BC in SD rats. (A) Histopathological (*i.e.*, H&E) and (B) immunofluorescence (*i.e.*, caspase-3 and Ki-67) analysis of tumor tissue sections after treatment with PBS as a control, free HCPT, or R_9_NG/HCPT. The scale bars in (A) and (B) represent 0.5 cm and 100 *μ*m, respectively. The quantitative analysis of (C) caspase-3 and (D) Ki-67 expression after treatments with different HCPT formulations. Data are presented as mean ± standard deviation (*n* = 3; ^∗^*P* < 0.05, ^∗∗∗^*P* < 0.001).
