# Supplementary material for: Synergistically Enhanced Mucoadhesive and Penetrable Polypeptide Nanogel for Efficient Drug Delivery to Orthotopic Bladder Cancer
Source: Research (Wash D C). 2020 Aug 3;2020:8970135. doi: 10.34133/2020/8970135 (PMC7420878; doi:10.34133/2020/8970135)
Supplement: Supplementary Materials — Supplementary 1. Materials and Methods. Supplementary 2. Figure S1: 1H NMR spectrum of aPEG–P(LP-co-LC) in deuterated trifluoroacetic acid (TFA-d). Supplementary 3. Figure S2: 1H NMR spectrum of t-Boc-NH-PEG–P(LP-co-LC) in deuterated trifluoroacetic acid (TFA-d). Supplementary 4. Figure S3: 1H NMR spectrum of NH2-PEG–P(LP-co-LC) in deuterated trifluoroacetic acid (TFA-d). Supplementary 5. Figure S4: 1H NMR spectra of (A) R9-PEG–P(LP-co-LC), (B) Mal-PEG–P(LP-co-LC), and (C) R9C in deuterated trifluoroacetic acid (TFA-d). Supplementary 6. Figure S5: FCM analysis calculated apoptotic cell populations after co-incubating human BC 5637 cells with PBS as a control, free HCPT, NG/HCPT, or R9NG/HCPT for 48 h. The lower-left (Q3), lower-right (Q4), upper-right (Q1), and upper-left (Q2) quadrants in each panel indicated the populations of healthy, early and late apoptotic, and necrotic cells, respectively. Supplementary 7. Figure S6: In vivo biodistribution of free HCPT and R9NG/HCPT. Data are presented as mean ± standard deviation (n = 3; ∗∗P < 0.01). Supplementary 8. Figure S7: In vivo anti-tumor efficacy on orthotopic BC in SD rats. (A) Cystography and (B) external and internal surface of bladders after intravesical chemotherapy with PBS as a control, free HCPT, or R9NG/HCPT. (C) Evolution of body weight and (D) survival rate during the experiments. The scale bars represent (A) 1.0 cm and (B) 0.5 cm, respectively. Data are presented as mean ± standard deviation (n = 8). Supplementary 9. Figure S8: histopathology and immunofluorescence of orthotopic BC in SD rats. (A) Histopathological (i.e., H&E) and (B) immunofluorescence (i.e., caspase-3 and Ki-67) analysis of tumor tissue sections after treatment with PBS as a control, free HCPT, or R9NG/HCPT. The scale bars in (A) and (B) represent 0.5 cm and 100 μm, respectively. The quantitative analysis of (C) caspase-3 and (D) Ki-67 expression after treatments with different HCPT formulations. Data are presented as mean ± standard [file 8970135.f1.doc]

Supplementary Materials

**Synergistically Enhanced Mucoadhesive and Penetrable Polypeptide Nanogel for Efficient Drug Delivery to Orthotopic Bladder Cancer**

Hui Guo^1,2^, Faping Li^2^, Heping Qiu^1,2^, Weiguo Xu^1^, Pengqiang Li^1^, Yuchuan Hou^2^, Jianxun Ding^1^,*, and Xuesi Chen^1^

*^1^Key Laboratory of Polymer Ecomaterials, Changchun Institute of Applied Chemistry, Chinese Academy of Sciences, Changchun 130022, P. R. China*

*^2^Department of Urinary Surgery, The First Hospital of Jilin University, Changchun 130021, P. R. China*

*Correspondence should be addressed to Jianxun Ding; jxding@ciac.ac.cn

**Materials and Methods**

*Materials.* L-phenylalanine and L-cystine were supplied by GL Biochem Co., Ltd. (Shanghai, P. R. China). L-phenylalanine *N*-carboxyanhydride (LP NCA) and L-cystine *N*-carboxyanhydride (LC NCA) were synthesized as described in our previous work with slight modification [1]. Allyloxy poly(ethylene glycol) (aPEG) of 3400 g mol^−1^ in number-average molecular weight (*M*_n_) was purchased from Haian Petrochemical Plant (Haian, P. R. China). Acetic acid, ammonium acetate, triethanolamine, and other chemicals were purchased from Sinopharm Chemical Reagent Co., Ltd. (Shanghai, P. R. China) and used as received. Cysteine-modified oligoarginine (R_9_C) was purchased from Shanghai Qiangyao Biotechnology Co., Ltd. (Shanghai, P. R. China). 10-Hydroxycamptothecin (HCPT) was purchased from Beijing Huafeng United Technology Co., Ltd. (Beijing, P. R. China).

Tissue culture polystyrene 6-well and 96-well plates were obtained from Corning Costar Co. (Cambridge, MA, USA). Dulbecco's modified Eagle's medium (DMEM) and fetal bovine serum (FBS) were bought from Gibco (Grand Island, NY, USA). Methyl thiazolyl tetrazolium (MTT) and 4′,6-diamidino-2-phenylindole dihydrochloride (DAPI) were purchased from Sigma-Aldrich (Shanghai, P. R. China). *N*-Methyl-*N*-nitrosourea (MNU) was purchased from Sichuan Hainuowei Technology Co., Ltd. (Sichuan, P. R. China). 22-Gauge closed IV catheter systems were purchased from Suzhou Bidi Medical Devices Co., Ltd. (Suzhou, P. R. China). All the other solvents and reagents were sourced from Sigma-Aldrich (Shanghai, P. R. China) and used as received. The purified deionized water was prepared by the Milli-Q plus system (Millipore Co., Billerica, MA, USA).

*Preparation of Oligoarginine-Poly(ethylene glycol)−Poly(L-phenylalanine-co-L-cystine) (R_9_-PEG−P(LP-co-LC)).* R_9_-PEG−P(LP-*co*-LC) was synthesized by the ring-opening polymerization (ROP) of LP NCA and LC NCA with the amino-terminated aPEG (aPEG-NH_2_) as a macroinitiator, and the R_9_ was decorated at the end of the terminal of PEG by click reaction. In brief, aPEG-NH_2_, LP NCA, and LC NCA were dissolved in anhydrous *N*,*N*-dimethylformamide (DMF) in a flame-dry flask. The reaction mixture was stirred at 25 °C for 72 h. The solution was then precipitated into an excess amount of diethyl ether. The obtained product was further washed twice with diethyl ether and dissolved in anhydrous DMF, and then *tert*-butyl (2-mercaptoethyl) carbamate was added. The solution was stirred for 4 h under the irradiation of 365 nm wavelength with the 2,2-dimethoxy-2-phenylacetophenone as a photoinitiator. The solution was stirred under a nitrogen atmosphere for another 48 h in the dark. The obtained product was then precipitated into an excess amount of diethyl ether.

The precipitate was dissolved in trifluoroacetic acid (TFA), and stirred at 25 °C for 1 h. Subsequently, the mixture was settled with excess ether, dialyzed, and freeze-dried to give a product of NH_2_-PEG−P(LP-*co*-LC). The obtained NH_2_-PEG−P(LP-*co*-LC) was then modified with 3-maleimidopropionic acid. The obtained product was Mal-PEG−P(LP-*co*-LC) and further dissolved in anhydrous DMF, then R_9_C was added, and the mixture was stirred for another 72 h. The mixture was dialyzed and freeze-dried to give a product of R_9_-PEG−P(LP-*co*-LC).

*Determination of Drug Loading Content and Drug Loading Efficiency.* The drug-loaded nanogel (*i.e.*, R_9_NG/HCPT) was prepared through a facile diffusion and dialysis method. In short, R_9_-PEG−P(LP-*co*-LC) (500.0 mg) and HCPT (200.0 mg) were dissolved in 20.0 mL of DMF by vortex and sonication. The mixture was then stirred for 2 h at room temperature. Then, 10.0 mL of deionized water was added to the solution while stirring, followed by dialysis for 24 h. The dialysis medium was refreshed five times, and the whole procedure was performed in the dark. Then, the solution was filtered and lyophilized to obtain R_9_NG/HCPT. The drug-loaded nanogel (*i.e.*, NG/HCPT) consisting of Mal-PEG−P(LP-*co*-LC) and HCPT was successfully prepared by the same procedure as a control.

To determine the drug loading content (DLC) and drug loading efficiency (DLE), the freeze-dried NG/HCPT or R_9_NG/HCPT was accurately weighed and dissolved in DMF. The HCPT concentration was then analyzed by ultraviolet−visible (UV−vis) spectrophotometry using a standard curve method (λ_abs_ = 365 nm). The DLC and DLE of NG/HCPT or R_9_NG/HCPT were calculated according to Equations (1) and (2), respectively.

$DLC (\%)=\frac{Amount of Drug in Nanogel}{Amount of Loading Nanogel}\times100\%$ (1)

$DLE (\%)=\frac{Amount of Drug in Nanogel}{Total Amount of Feeding Drug}\times100\%$ (2)

*Characterizations of R_9_NG/HCPT.* Proton nuclear magnetic resonance (^1^H NMR) spectra were recorded on a Bruker AV 400 NMR spectrometer (Ettlingen, Germany) with deuterated trifluoroacetic acid (TFA-*d*) as a solvent. The morphology of R_9_NG/HCPT was revealed by transmission electron microscopy (TEM) measurement on a JEM-1011 TEM (JEOL, Tokyo, Japan) with an accelerating voltage of 100 kV. To prepare the TEM sample, a small drop of R_9_NG/HCPT aqueous solution was deposited onto a 230 mesh copper grid coated with carbon, and allowed to dry at room temperature for over 24 h. The hydrodynamic radii (*R*_h_s) of NG/HCPT and R_9_NG/HCPT were determined by dynamic laser scattering (DLS) measurements on a WyattQELS instrument with a vertically polarized He−Ne laser (DAWN EOS, Wyatt Technology Co., Santa Barbara, CA, USA). The scattering angle was fixed at 90°. The samples were prepared in aqueous solution at a concentration of 100.0 μg mL^−1^. Before measurements, the solution was filtered through a 0.45 μm Millipore filter. The zeta potentials of NG/HCPT and R_9_NG/HCPT were determined by a Zeta Potential/BI-90Plus Particle Size Analyzer (Brookhaven, USA).

*In Vitro HCPT Release.* The *in vitro* HCPT release kinetics from different HCPT formulations were investigated in phosphate-buffered saline (PBS; pH 7.4, Tween-80 (0.1%, *W*/*V*)) without or with 5.0 or 10.0 mM DTT. Typically, 0.1 mg of free HCPT or 1.0 mg of freeze-dried NG/HCPT or R_9_NG/HCPT was dissolved in 10.0 mL of corresponding release medium and introduced into a dialysis bag (molecular weight cutoff (MWCO) = 3,500 Da). The release experiment was initiated by placing the end-sealed dialysis bag into 100.0 mL of release medium at 37 °C with a continuous shaking rate of 70 rpm. At predetermined time intervals, 2.0 mL of dialysate was taken out, and an equivalent amount of fresh medium was added. The sample solution was acidified with 1.0 N HCl, and 20.0 μL was measured for the accumulative amount of HCPT release by high-performance liquid chromatography (HPLC; λ_abs_ = 371 nm).

*Intracellular Drug Release.* To evaluate the cell uptake and intracellular release behaviors of R_9_NG/HCPT, human bladder cancer (BC) 5637 cells were used. Confocal laser scanning microscopy (CLSM, LSM 780, Carl Zeiss, Jena, Germany) was performed to assess the cell uptake of R9NG/HCPT qualitatively. Human BC 5637 cells were seeded onto glass coverslips in 6-well plates at a density of 1.5 × 10^5^ cells in 2.0 mL of complete DMEM containing 10% (*V*/*V*) FBS, supplemented with 50 IU mL^−1^ penicillin and 50 IU mL^−1^ streptomycin per well, and cultured at 37 °C in 5% (*V*/*V*) carbon dioxide (CO_2_) atmosphere for 24 h. Then the incubation medium was removed, and free HCPT, NG/HCPT, or R_9_NG/HCPT in 2.0 mL of complete DMEM was added with a final HCPT concentration of 1.25 μg mL^−1^. At predetermined time intervals, the culture media were removed, and the cells were washed thrice with PBS. After that, the cells were fixed with 4% (*W/V*) PBS-buffered formaldehyde for 20 min at room temperature. Then, the cells were washed thrice with PBS. The representative microimages of cells were measured on CLSM.

The cell uptake and intracellular release behaviors were further confirmed quantitatively by microplate reader as described by Wei *et al.* [2]. The cells were cultured in 96-well plates and co-incubated with different HCPT formulations (1.25 μg mL^−1^) for the desired period, and then washed thrice with ice-cold PBS. Cell lysis solution (1% (*V*/*V*) Triton X-100) was used to treat the samples, which was followed by the addition of sodium hydroxide (NaOH) to solute the internalized HCPT. After these treatments, the plate was measured at 384 nm by an Infinite M200 microplate spectrophotometer (Tecan, Durham, USA). Each experiment was performed in triplicate.

*In Vitro Cell Proliferation Inhibition Assays.* To assess the potential cytotoxicity of R_9_NG/HCPT, a standard MTT assay was carried out. The cells were planted into 96-well plates at 1.0 × 10^4^ cells per well in 200.0 μL of complete DMEM and incubated at 37 °C for 24 h. Then, the incubation medium was removed, and free HCPT, NG/HCPT, or R_9_NG/HCPT in 200.0 μL of complete DMEM with various HCPT concentrations (0.08 – 10.0 μg mL^−1^) was added. The cells without pretreatment were used as a control. The cells were subjected to MTT assay after being co-incubated for another 24 h. The absorbance of the solution was measured at 490 nm on a Bio-Rad 680 microplate reader (Bio-Rad Laboratories, Hercules, CA, USA). The cell viability (%) was calculated according to Equation 3.

(3)

In Equation (3), *A*_sample_ and *A*_control_ represented the absorbances of the sample well and control well, respectively.

*Apoptosis Detection.* The HCPT-induced apoptosis of human BC 5637 cells was assessed by flow cytometry analysis (FCM). Human BC 5637 cells were seeded in 6-well plates at a density of 2.0 × 10^5^ per well and incubated for 24 h. Subsequently, the incubation medium was removed, and free HCPT, NG/HCPT, or R_9_NG/HCPT in 2.0 mL of complete DMEM was added with an HCPT concentration of 0.1 μg mL^−1^. The cells without pretreatment were used as a control. Cells were co-incubated for another 24 or 48 h at 37 °C and harvested with ethylenediaminetetraacetic acid (EDTA)-free trypsin, centrifuged at 1,000 rpm for 5 min and washed consecutively with PBS. Then the cells were resuspended in 0.5 mL binding buffer stained with 5.0 μL of Annexin V-FITC at room temperature for 10 min, followed by the addition of 5.0 μL propidium iodide (PI) on ice. At the end of co-incubation, the cells were analyzed immediately by FCM. The first 10,000 events were acquired by CXP analysis software V2.1 (Applied Cytometry Systems, Dinnington, UK).

*Animal Procedures.* The male C57Bl/6 mice weighing 18 − 20 g and Sprague-Dawley (SD) rats weighing 170 − 200 g were purchased from the Laboratory Animal Center of Jilin University (Changchun, P. R. China). All animals received care in compliance with the guidelines outlined in the Guide for the Care and Use of Laboratory Animals, and all procedures were approved by the Animal Care and Use Committee of Jilin University.

*BC Model Induction and Histopathology Determination.* The orthotopic BC model in mouse was established by instilling MB49 cells into the bladders that had been pretreated with poly(L-lysine) (PLL) solution. Briefly, PLL (100.0 μL of 0.1 mg mL^−1^ for 20 min) was used to disrupt the glycosaminoglycan layer. 50.0 μL of MB49 cell suspension (1.0 × 10^5^ cells) were slowly injected into the bladder of C57Bl/6 mice and allow the cells to dwell in the bladder for 50 min [3]. The orthotopic BC model in rats was induced by intravesical instillation of MNU. Briefly, the rats were anesthetized with ether inhalation and then received 2.0 mg of MNU dissolved in 0.2 mL of sodium citrate buffer *via* a lubricated 22-gauge angiocatheter every other week for a total of eight weeks [4]. To avoid spontaneous micturition, the rats remained anesthetized for approximately 2 h after catheterization. The rats were observed once a day, weighed weekly, and palpated for the bladder lesions every other day. At the end of the MNU treatment, two rats were sacrificed randomly, and the bladders were harvested, fixed in 4% (*W/V*) PBS-buffered paraformaldehyde, and followed by dehydration, clearing, wax infiltration, and embedding. The sections of these embedded tissue samples were stained with hematoxylin and eosin (H&E) to assess the incidence and progressions of BC by microscope (Nikon TE2000U, Kanagawa, Japan).

*Mucoadhesiveness and Penetrability Studies.* The mucoadhesiveness and penetrability of R_9_NG/HCPT were investigated by CLSM. First, the male SD rats with BC were anesthetized, and then the animals received an intravesical instillation of free HCPT or R_9_NG/HCPT solution with an equivalent HCPT concentration of 6.0 mg per kg body weight (mg (kg BW)^−1^) *via* a lubricated 22-gauge closed IV catheters system. The rats were anesthetized for approximately 2 h and monitored carefully after catheterization. At predetermined time intervals, the bladders were excised, opened, and washed five times with PBS. For the mucoadhesiveness study, tissue sections were prepared by cutting flattened bladder into the square and washed thoroughly with PBS again. The urothelial surfaces of these prepared bladder sections were determined by CLSM immediately. For the penetrability study, the flattened bladders were embedded in Tissue-Tek O.C.T. Compound embedding medium (Miles Inc., Diagnostics Division, Elkhart, IN, USA). Then, cryogenic sections of 6 μm thickness were sliced from the mucous membrane to the serous membrane serially using a freezing microtome (Leica CM 1900, Wetzlar, Germany). CLSM then observed the sections.

*In Vivo Biodistribution.* The *in vivo* biodistribution of R_9_NG/HCPT was conducted by high-performance liquid chromatography (HPLC). Male SD rats with BC were treated with free HCPT or R_9_NG/HCPT at an equivalent HCPT dose of 6.0 mg (kg BW)^−1^ by intravesical instillation (*n* = 3 for each group) and sacrificed 6 h later. The bladder and tested organs (*i.e*., the heart, liver, spleen, lung, and kidney) were excised, washed, and accurately weighted. The tissue samples were homogenized with normal saline, which was acidified to pH 3.0 with acetic acid. Afterward, the tissue homogenates were extracted with two volumes of a cold mixture of methanol/acetonitrile (1/1, *V/V*). The clear suspensions were obtained by centrifugation at 15,000 rpm at 4 °C for 10 min, and 20 μL of that was used to determine the concentration of HCPT by HPLC. The mobile phase was a mixture of acetonitrile/aqueous buffer (67/33, *V/V*), in which the aqueous buffer contained 75.0 mmol L^−1^ ammonium acetate, 5.0 mmol L^−1^ triethylamine, and 0.5% (*V/V*) acetic acid [5,6].

*In Vivo Anti-tumor Efficacy.* A total of 24 C57Bl/6 mice with BC were randomly divided into four groups: PBS, free HCPT, NG/HCPT, and R_9_NG/HCPT (*n* = 6). Different HCPT formulations were administered weekly by catheterization at a dosage of 6.0 mg (kg BW)^−1^ for a total of four treatments. The anti-tumor efficiency of R_9_NG/HCPT was further evaluated in the orthotopic BC model in rats. The tumor-bearing rats were randomly divided into three groups, each consisted of eight rats. The animals were treated with PBS, free HCPT, and R_9_NG/HCPT at an equivalent HCPT dose of 6.0 mg (kg BW)^−1^ by intravesical instillation weekly for a total of six treatments. Cystography was used once a week for monitoring the tumor size and the progress of BC. The body weight was measured at the same time as an indicator of systemic toxicity.

*Histological and Immunofluorescence Analysis.* One week after the last intravesical instillation of various HCPT formulations, the experimental animals were sacrificed, and the bladders were excised, fixed in 4% (*W/V*) PBS-buffered paraformaldehyde, and followed by dehydration, clearing, wax infiltration, and embedding. The sections of these embedded tissue samples were used for H&E staining. Moreover, the detection of apoptotic DNA fragments was performed using the terminal deoxyribonucleotidyl transferase (TdT)-mediated biotin-16-dUTP nick-end labeling (TUNEL) assay. Some other sections were stained with the immunofluorescence method to assess the expression of caspase-3 (Abcam, Cambridge, MA, USA) and Ki-67 (Abcam, Cambridge, MA, USA). The alterations of histology were evaluated by microscope. The immunofluorescence staining results were examined by CLSM.

*Statistical Analysis.* All experiments were performed at least thrice. The results were presented as means ± standard deviation. Data were analyzed for statistical significance using Student's *t*-test. **P* < 0.05 was considered statistically significant, and ***P* < 0.01 and ****P* < 0.001 were considered highly significant.

**References**

[1] J. Chen, J. Ding, Y. Wang, *et al.*, "Sequentially responsive shell-stacked nanoparticles for deep penetration into solid tumors," *Advanced Materials*, vol. 29, no. 32, article 1701170, 2017.

[2] W. Wei, Z.G. Yue, J.B. Qu, *et al.*, "Galactosylated nanocrystallites of insoluble anticancer drug for liver-targeting therapy: An *in vitro* evaluation," *Nanomedicine*, vol. 5, no. 4, pp. 589–596, 2010.

[3] A.J. Vandeveer, J.K. Fallon, R. Tighe, *et al.*, "Systemic immunotherapy of non-muscle invasive mouse bladder cancer with avelumab, an anti-PD-L1 immune checkpoint inhibitor," *Cancer Immunology Research*, vol. 4, no. 5, pp. 452–462, 2016.

[4] Q. Pan, G.L. Yang, J.H. Yang, *et al.*, "Metformin can Block Precancerous Progression to Invasive Tumors of Bladder through Inhibiting Stat3-Mediated Signaling Pathways," *Journal of Experimental and Clinical Cancer Research*, vol. 34, article 77, 2015.

[5] L. Zhang, M. Yang, Q. Wang, *et al.*, "10-Hydroxycamptothecin loaded nanoparticles: Preparation and anti-tumor activity in mice," *Journal of Controlled Release*, vol. 119, no. 2, pp. 153–162, 2007.

[6] P. Li, Y. Zheng, H. Ran, *et al.*, "Ultrasound triggered drug release from 10-hydroxycamptothecin-loaded phospholipid microbubbles for targeted tumor therapy in mice," *Journal of Controlled Release*, vol. 162, no. 2, pp. 349–354, 2012.


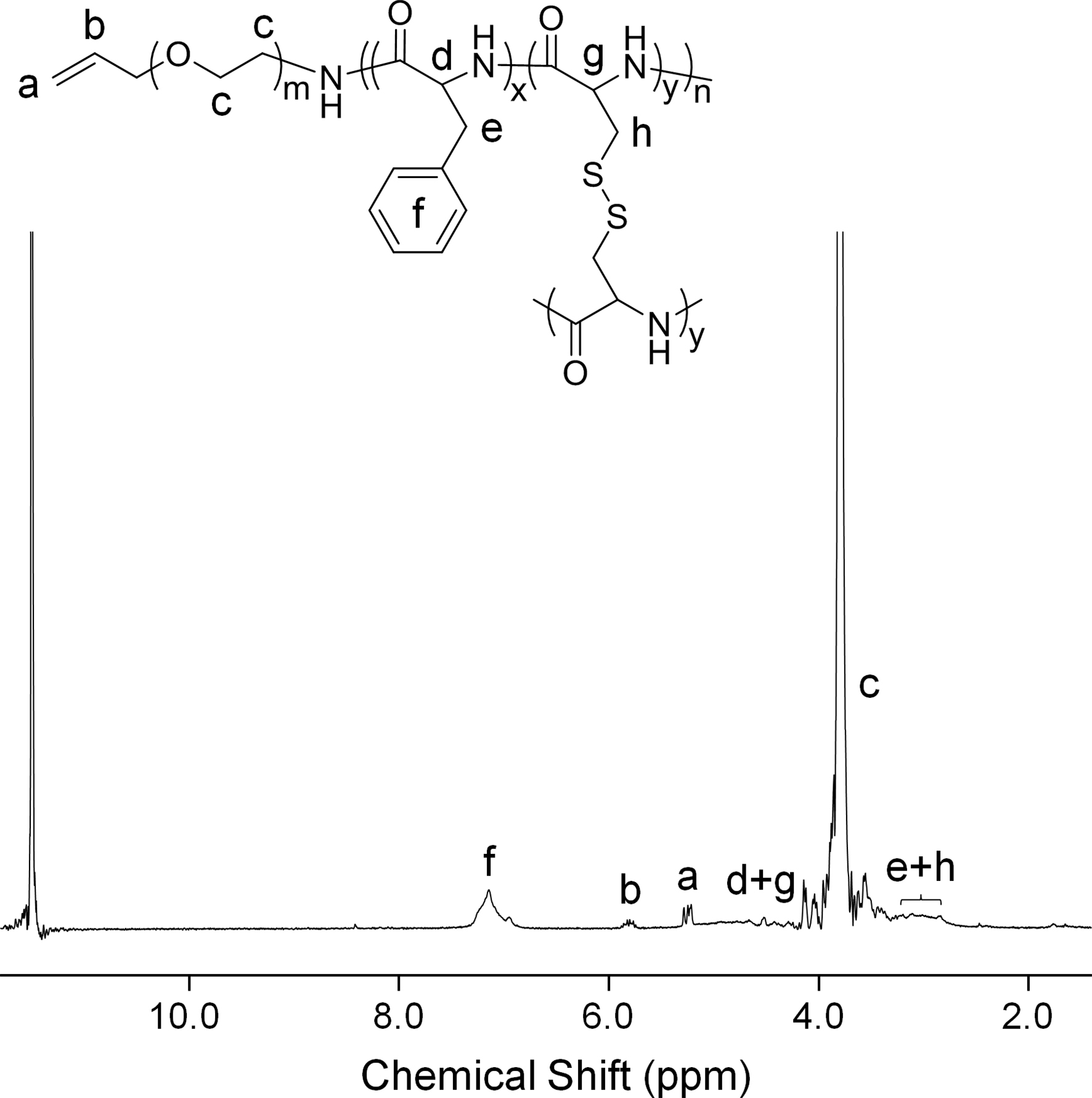


FIGURE S1: ^1^H NMR spectrum of aPEG−P(LP-*co*-LC) in TFA-*d*.


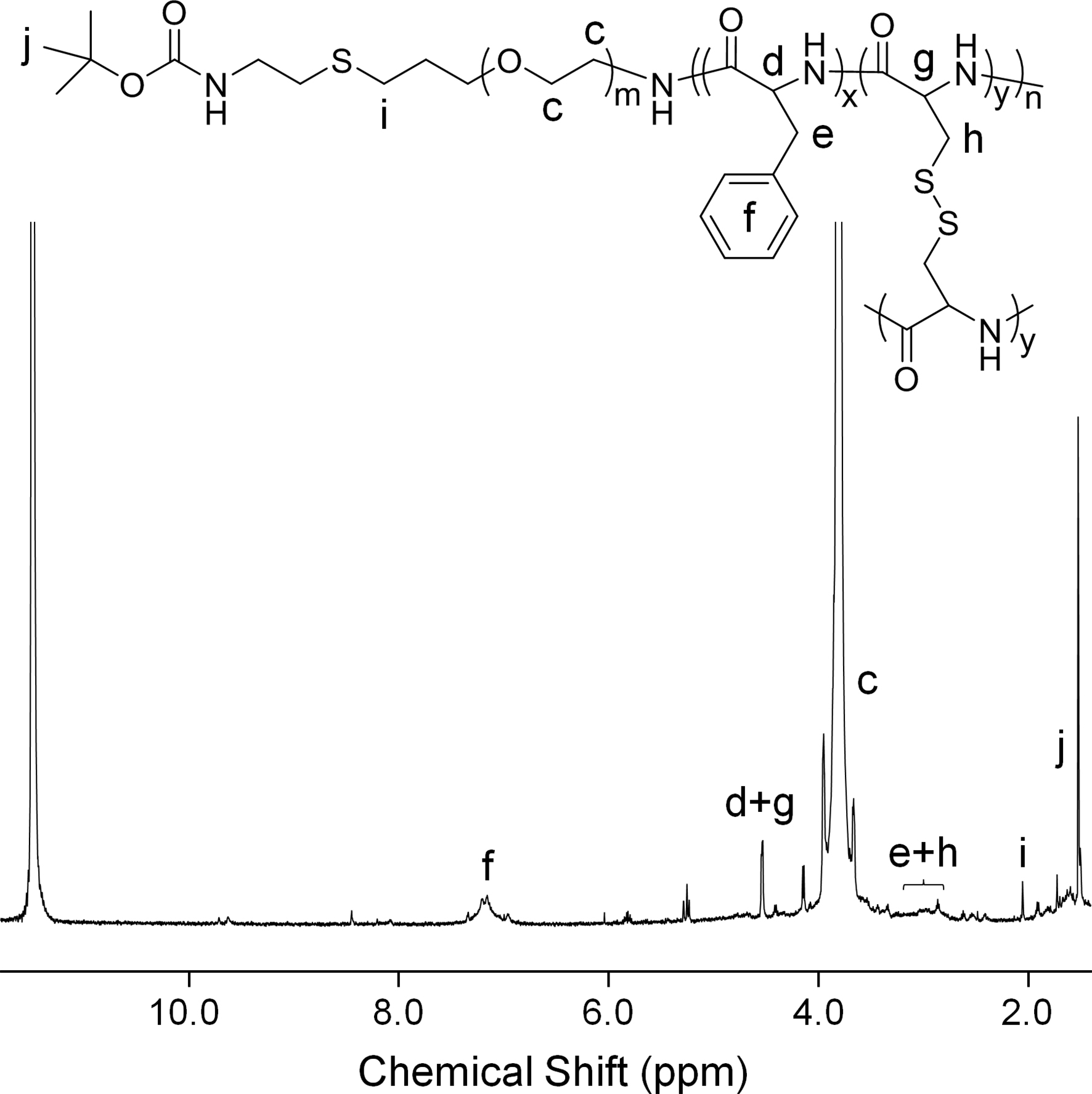


FIGURE S2: ^1^H NMR spectrum of *t*-Boc-NH-PEG−P(LP-*co*-LC) in TFA-*d*.


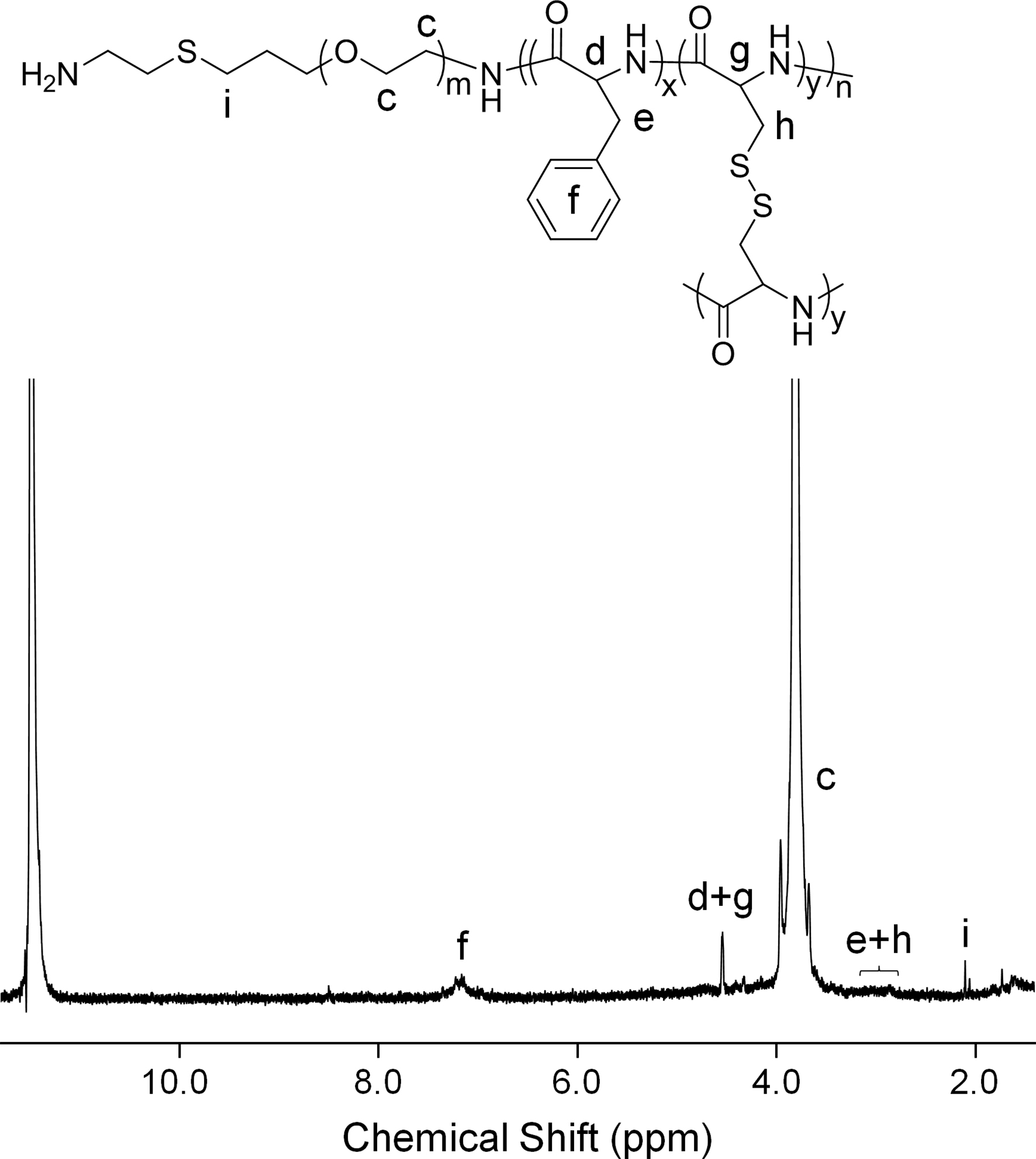


FIGURE S3: ^1^H NMR spectrum of NH_2_-PEG−P(LP-*co*-LC) in TFA-*d*.


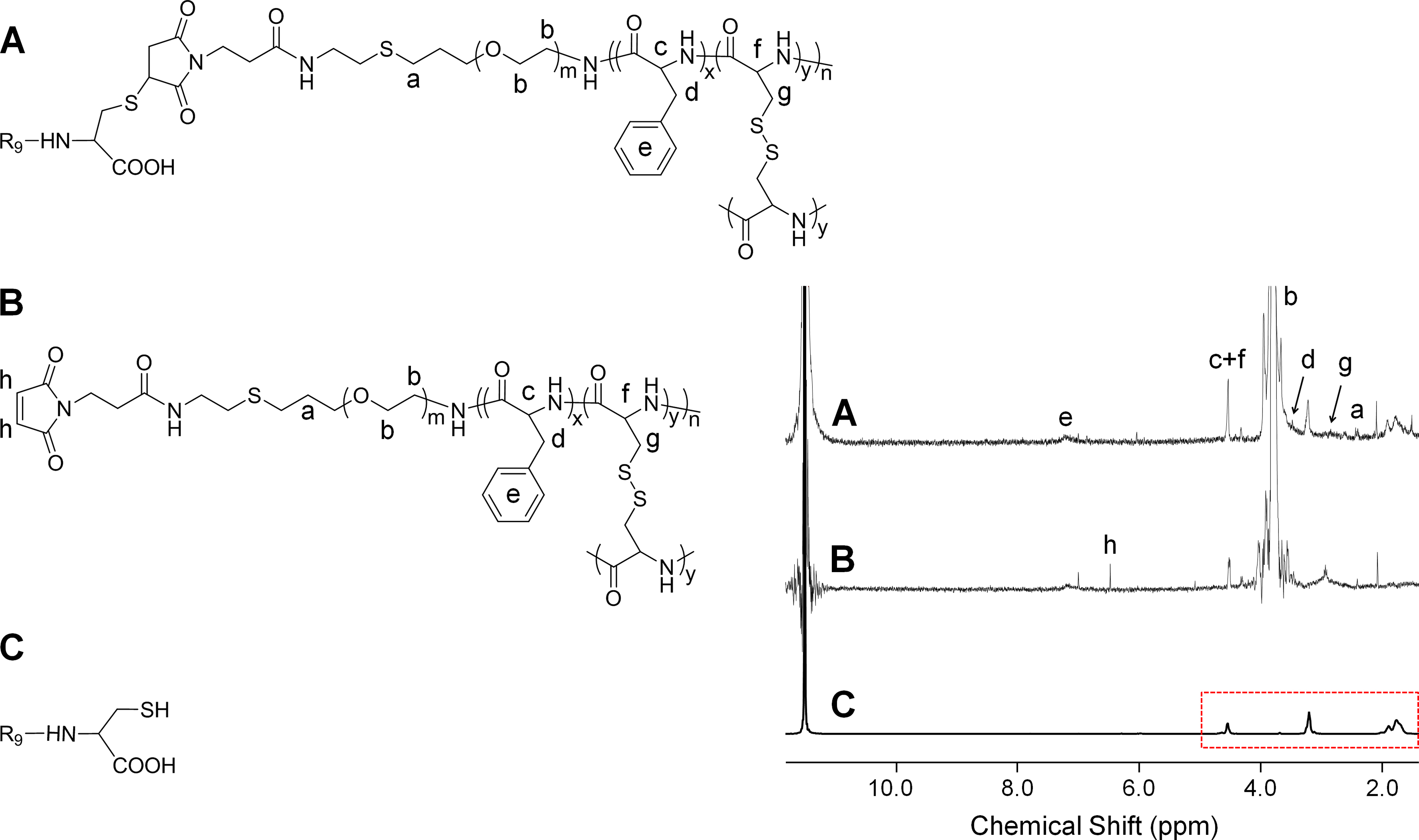


FIGURE S4: ^1^H NMR spectra of (A) R_9_-PEG−P(LP-*co*-LC), (B) Mal-PEG−P(LP-*co*-LC), and (C) R_9_C in TFA-*d*.


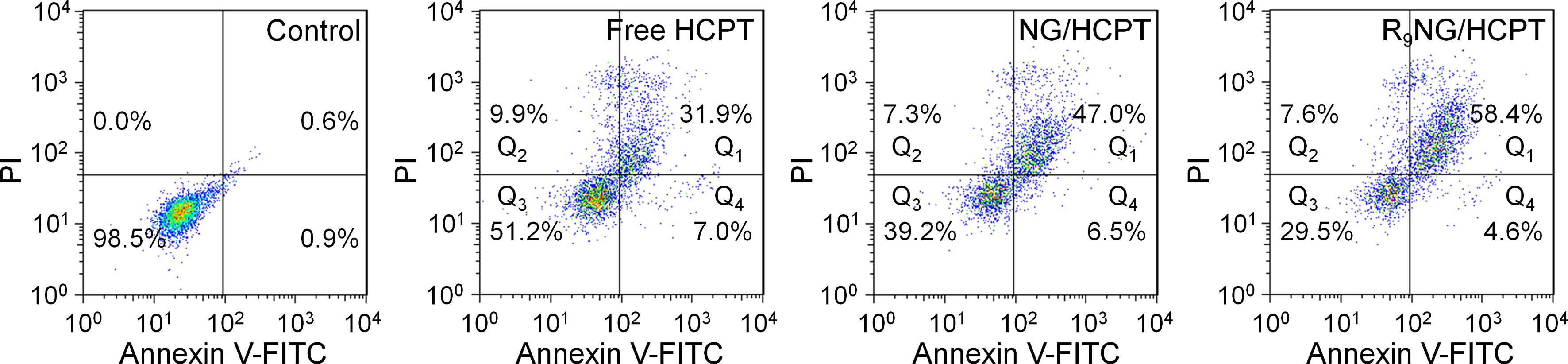


FIGURE S5: Apoptotic cell populations were calculated by FCM analysis after co-incubating human BC 5637 cells with PBS as a control, free HCPT, NG/HCPT, or R_9_NG/HCPT for 48 h. The lower-left (Q3), lower-right (Q4), upper-right (Q1), and upper-left (Q2) quadrants in each panel indicated the populations of normal, early and late apoptotic, and necrotic cells, respectively.


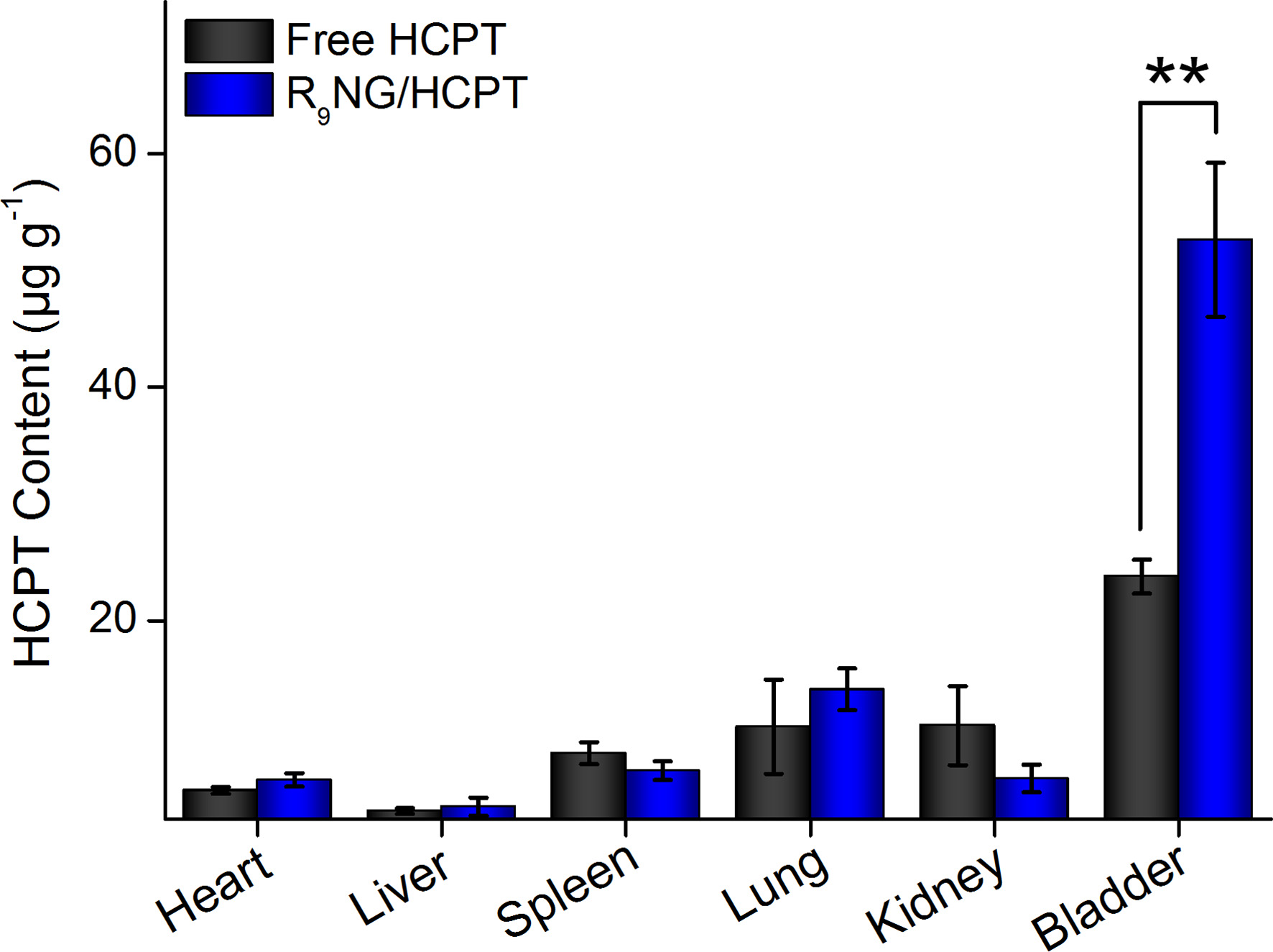


FIGURE S6: *In vivo* biodistribution of free HCPT and R_9_NG/HCPT. Data are presented as mean ± standard deviation (*n* = 3; ***P* < 0.01).


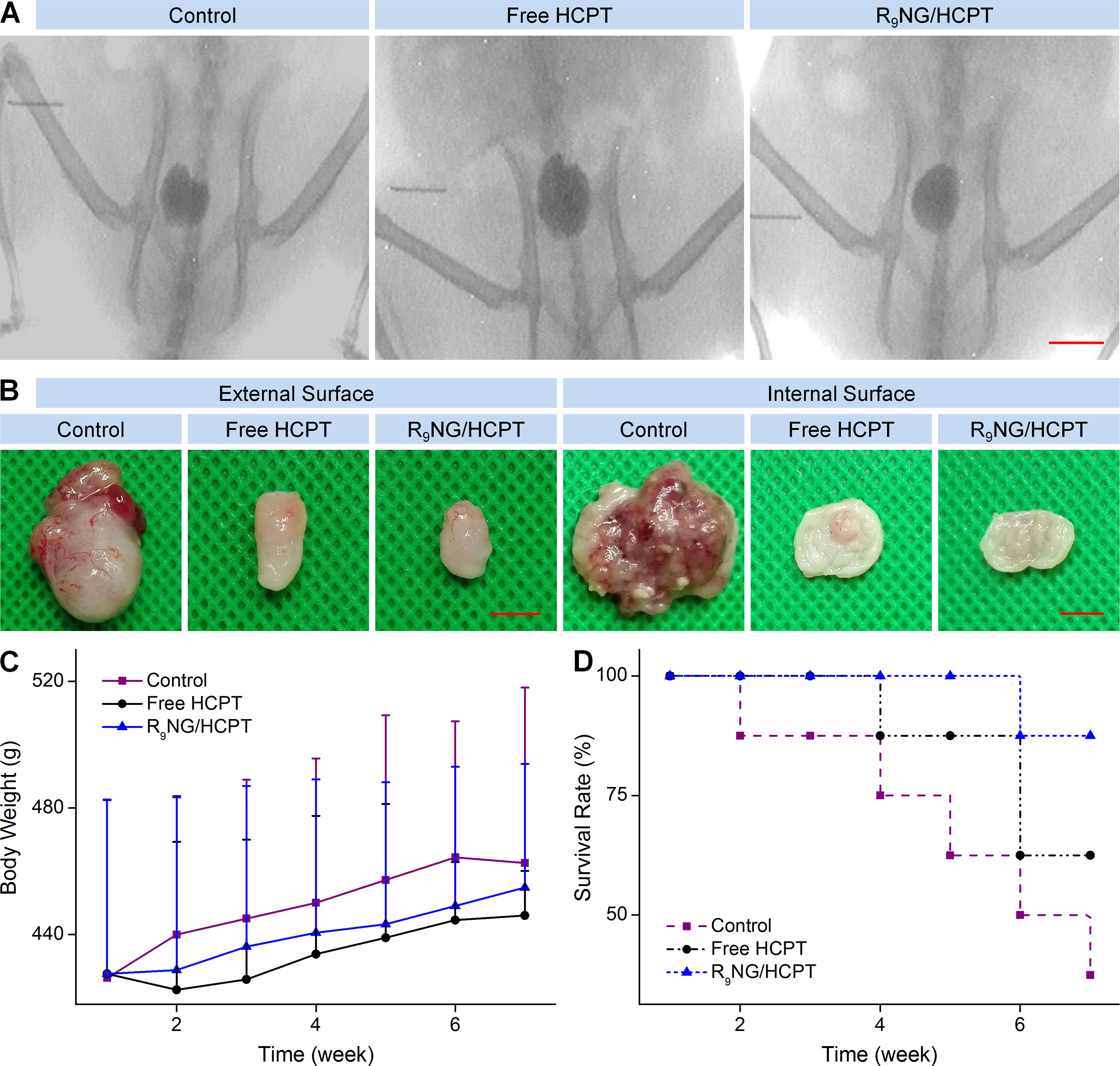


FIGURE S7: *In vivo* anti-tumor efficiency on orthotopic BC in SD rats. (A) Cystography and (B) external and internal surface of bladders after intravesical chemotherapy with PBS as a control, free HCPT, or R_9_NG/HCPT. (C) Evolution of body weight and (D) survival rate during the experiments. The scale bars represent (A) 1.0 cm and (B) 0.5 cm, respectively. Data are presented as mean ± standard deviation (*n* = 8).


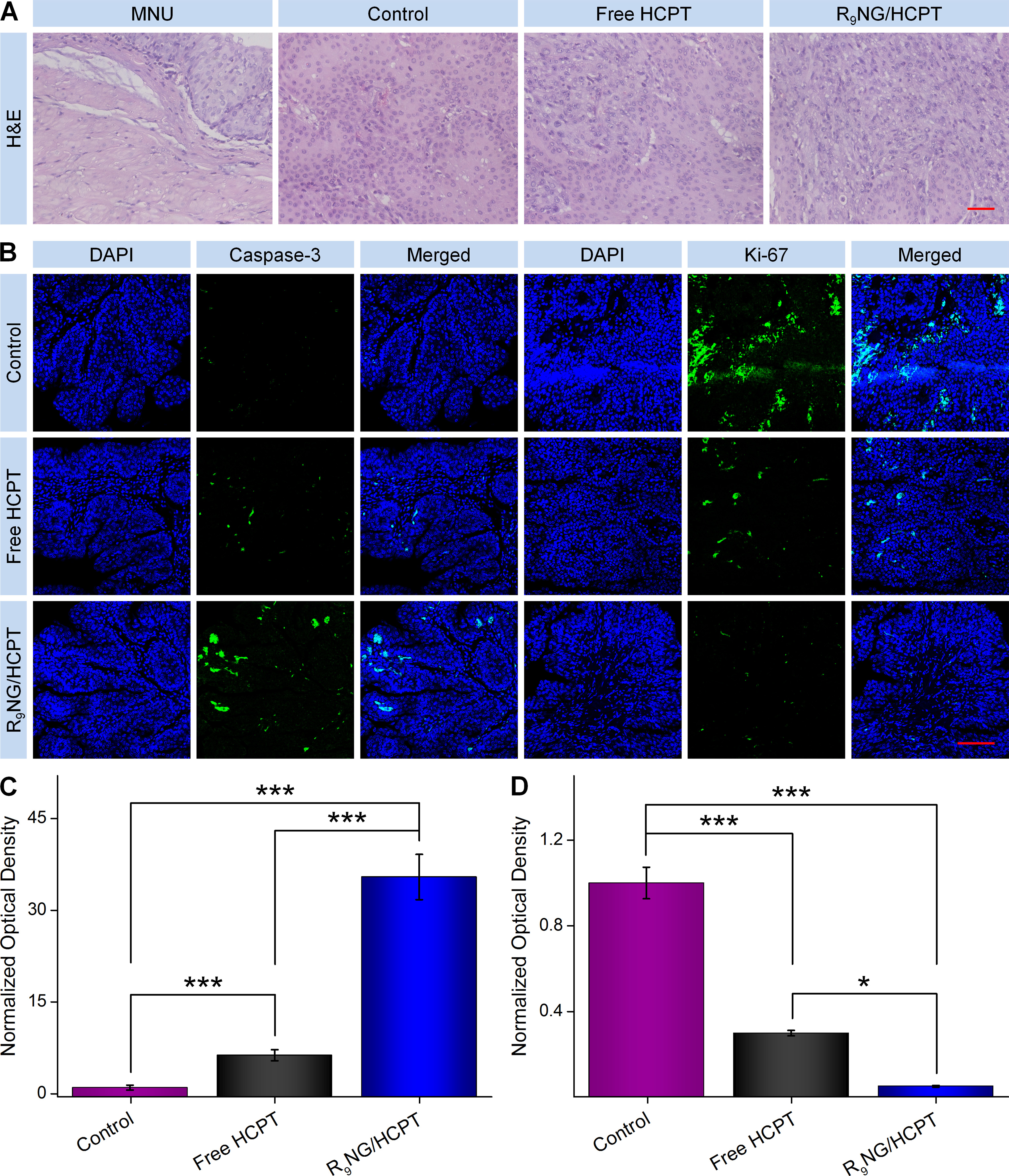


FIGURE S8: Histopathology and immunofluorescence of orthotopic BC in SD rats. (A) Histopathological (*i.e.*, H&E) and (B) immunofluorescence (*i.e*., caspase-3 and Ki-67) analysis of tumor tissue sections after treatment with PBS as a control, free HCPT, or R_9_NG/HCPT. The scale bars in (A) and (B) represents 0.5 cm and 100 μm, respectively. The quantitative analysis of (C) caspase-3 and (D) Ki-67 expression after treatment with different HCPT formulations. Data are presented as mean ± standard deviation (*n* = 3; **P* < 0.05, ****P* < 0.001).
